# Understanding the Origin of the Hysteresis of High-Performance Solution Processed Polycrystalline SnO_2_ Thin-Film Transistors and Applications to Circuits

**DOI:** 10.3390/membranes12010007

**Published:** 2021-12-22

**Authors:** Christophe Avis, Jin Jang

**Affiliations:** Display Research Center, Department of Information Display, Kyung Hee University, Seoul 02447, Korea; jjang@khu.ac.kr

**Keywords:** polycrystalline oxide semiconductor, tin oxide, thin film transistor, hysteresis, gate bias stress, high k, high bandgap, reliability, device application

## Abstract

Crystalline tin oxide has been investigated for industrial applications since the 1970s. Recently, the amorphous phase of tin oxide has been used in thin film transistors (TFTs) and has demonstrated high performance. For large area electronics, TFTs are well suited, but they are subject to various instabilities due to operating conditions, such as positive or negative bias stress PBS (NBS). Another instability is hysteresis, which can be detrimental in operating circuits. Understanding its origin can help fabricating more reliable TFTs. Here, we report an investigation on the origin of the hysteresis of solution-processed polycrystalline SnO_2_ TFTs. We examined the effect of the carrier concentration in the SnO_2_ channel region on the hysteresis by varying the curing temperature of the thin film from 200 to 350 °C. Stressing the TFTs characterized further the origin of the hysteresis, and holes trapped in the dielectric are understood to be the main source of the hysteresis. With TFTs showing the smallest hysteresis, we could fabricate inverters and ring oscillators.

## 1. Introduction

With the development of amorphous oxide semiconductor (AOS) electronics, various oxide-based semiconductors have been investigated. Indium gallium zinc oxide (IGZO) [1], indium zinc oxide (IZO) [2], indium gallium oxide (IGO) [3], zinc tin oxide (ZTO) [4], and indium zinc tin oxide (IZTO) [5] all have in common an amorphous phase, a conduction through s orbitals, optical transmittance of ~80% in the visible region, and offer a mobility of ~10 cm^2^/Vs [1].

Single cation-based oxides demonstrate a polycrystalline phase with higher mobilities. Zinc oxide (ZnO) [6], indium oxide (In_2_O_3_) [3], and tin oxide (SnO_2_) [7] are among the most investigated materials. One of the most valuable metrics in TFTs is the mobility, and ZnO TFTs have demonstrated 40–70 cm^2^/Vs [6,8] while In_2_O_3_ TFTs [4] have demonstrated mobilities ~50 cm^2^/Vs. Even the crystalline form of IGZO (c-axis crystalline IGZO, the so called CAAC-IGZO) has attracted attention due to the TFTs reaching mobilities ~90 cm^2^/Vs [9,10]. For a few years now, tin oxide has regained attention. Devices comprising SnO_2_ have demonstrated high mobility. Perovskite solar cells [11,12] and TFTs have been the main focus of research. TFTs have shown mobilities ranging from 40 to 147 cm^2^/Vs [13,14]. Even more recently, the amorphous phase of tin oxide [15,16] has demonstrated possible use in TFTs, reaching similar performances as the polycrystalline counterpart. Interestingly, in all these studies authors used a high-k dielectric as the gate insulator (ZrO_2_, HfO_2_, Al_2_O_3_)_,_ and a very thin channel layer (less than 10 nm).

With the use of high-k dielectrics [17], it is possible to obtain clockwise or anticlockwise hysteresis in the TFTs. The reasons are multiple, but shortly, in n-type based TFTs, the clockwise hysteresis can be resulting from the semiconductor (trapping of the electrons), while the anticlockwise from the gate dielectric (due to the movement of mobile ions for example) [18]. The anticlockwise behavior can be used in memory devices, while it can be detrimental for circuits. To fully understand the origin of the hysteresis (i.e., trapping of charge carriers, movement of mobile ions in the semiconductor or in the dielectric), measuring the hysteresis at slow and fast rates can help discriminate the origin.

We previously demonstrated that solution processed SnO_2_ using SnCl_2_ precursors could have various phases, and that the curing and annealing temperatures could impact significantly the various optical, electrical, and physical properties [19]. Here, we implemented the polycrystalline SnO_2_ thin films fabricated at various T_curing_ temperatures (200, 280, and 350 °C) and a fixed annealing temperature (T_anneal_ = 350 °C). High mobility TFTs (over 100 cm^2^/Vs) are obtained and we studied the hysteresis behaviors of the polycrystalline SnO_2_ TFTs (poly-SnO_2_ TFTs). By varying the sweep rate and applying negative and positive bias stresses to the TFTs, we can clearly identify the origin of the hysteresis.

## 2. Materials and Methods

### 2.1. Fabrication of the Precursor Solutions

Solutions of HfO_2_ (SnO_2_) were made by mixing HfCl_4_ (SnCl_2_) into a mixture of acetonitrile (Ac) and ethyleneglycol (Etg). We used 35% of Ac and 65% of Etg in volume%. The HfO_2_ (SnO_2_) precursor solutions were stirred in a N_2_ environment for 2 h (24 h) before use. The HfO_2_ precursor solutions had a concentration of 0.2 M and the SnO_2_ ones had a concentration of 0.2 M for the thin films, and 0.167 M for the TFTs.

### 2.2. Thin Film Fabrication and Analysis

The SnO_2_ thin films were fabricated by spin-coating at 2000 rpm during 25 s. After spin-coating, the layer was subject to a curing at 100 °C for 5 min, and a second curing step at 200, 280, or 350 °C for 5 min. The coating was repeated once. We measured the Hall effect on a Ecopia HMS-3000. The samples had a van der Pauw configuration. The data was collected from 15 points. We used the K*α* line (1.54 Å) for X-ray diffraction measurement to evaluate the crystallinity of the thin films. The surface roughness was evaluated by atomic force microscopy (AFM) by using a XE-7 from Park systems. The optical properties were evaluated by using an Scinco S4100. We measured X-ray photospectroscopy (XPS) with a Nexsa from ThermoFisher Scientific, by using the Al-Ka at 1486.6 eV as the X-ray source. Calibration was made with the carbon peak at 284.8 eV.

### 2.3. Thin Film Transistor Fabrication and Analysis

We fabricated poly-SnO_2_ TFTs by first sputtering 40 nm Mo as the gate on glass. After patterning, we spin-coated the HfO_2_ film. The coating was made at 2000 rpm for 25 s. The layer was then subject to a curing at 250 °C for 5 min, and UV treatment during 90 s. The deposition, curing, and treatment were repeated to obtain a 95 nm thick HfO_2_ layer. The samples were then subject to annealing at 350 °C for 2 h in air. The precursor solution of SnO_2_ was spin-coated at 4000 rpm during 25 s, and followed a curing step explained in the previous paragraph. After patterning, the TFTs were annealed at 350 °C for 2 h in air. We created via holes, sputtered and patterned IZO as the source/drain electrodes. Finally, a hot-plate annealing at 300 °C for 2 h and another hot-plate annealing step at 350 °C for 1 h were performed.

We measured the TFTs IV curves with a 4156 C semiconductor parameter analyzer. The TFTs had a width W and a length L of 50 and 10 μm, respectively. We evaluated the field-effect mobility in the linear region

(1) $$\mu_{\mathrm{lin}}={\frac{\partial I_{\mathrm{DS}}}{\partial V_{\mathrm{GS}}}|}_{V_{\mathrm{DS}}=0.1V}\times L/(V_{\mathrm{DS}}\times W\times \mathrm{Cox})$$

where Cox is the HfO_2_ capacitance.

The threshold voltage was evaluated at W/L × 10^−10^ A. The slope was evaluated as

(2) $$S.S.=\frac{\partial V_{\mathrm{GS}}}{\partial logI_{\mathrm{DS}}}.$$

The various parameters were averaged over 25 TFTs. The various parameters are extracted from the transfer curve measured at fast measurement rate, from negative to positive voltage. The hysteresis was measured at V_DS_ = 0.1 V, with a fast and a slow measurement rate related to an integration time of 6.04, and 20 ms, respectively. The positive (negative) bias stress PBS (NBS) were measured by applying V_GS_ = 3 V (−3 V) during 1 h. We note that the capacitance of the hafnium oxide dielectric was 219 nF/cm^2^ [15].

### 2.4. Circuit Fabrication

The inverter and ring oscillator followed the same process steps as the TFTs. The inverter had a load TFT with width and length of 50 and 6µm, respectively. The driving TFT had a width and length of 400 µm and 6 µm. The inverter had a depletion mode structure with the gate of the load TFT connected to the output.

The ring oscillator consisted of 11 of these inverters. The output of one is connected as the input of the following one. The last inverter being connected to the first inverter. A buffer inverter is put at the end of the ring oscillator to stabilize the measured output.

## 3. Results

### 3.1. Thin Film Analysis

**Figure 1:**
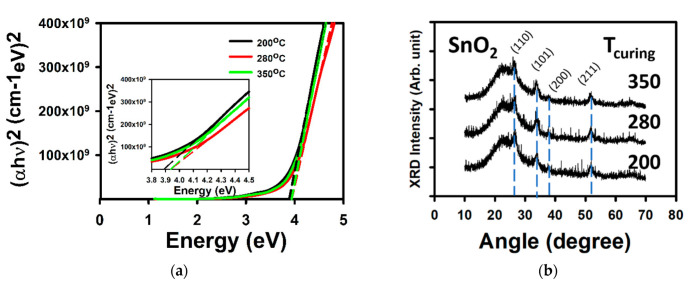
Thin film properties of solution processed SnO_2_ cured at 200, 280, and 350 °C, and annealed at 350 °C. (**a**) The Tauc plot for the extraction of the optical bandgap. The inset shows the energy between 3.8 and 4.5 eV. (**b**) XRD patterns. The dashed lines with the respective colors show the extraction of the bandgap. The dashed blue lines in (**b**) are here to help identify the various positions of the main peaks.

Figure 1 shows the optical and crystalline properties of SnO_2_. Figure 1a shows the extraction of the band gap from the Tauc plot [20]. The films with a T_curing_ of 200, 280, and 350 °C had a respective bandgap of 3.89, 3.94, and 3.94 eV. The films are adequate for application in invisible electronics [1]. Figure 1b shows the results of the XRD measurements. For all T_curing_, the thin films demonstrate the crystalline structure. Peaks related to the (110), (101), (200), and (211) are all observed. They are located at 26.6°, 33.8°, 37.8°, and 51.8°, respectively, for the thin film with the T_curing_ of 280 °C.

We extracted the carrier concentration N and the Hall mobility µ_H_ according to their definition:

(3) $${µ}_{H}=\frac{\left|R_{H}\right|}{\rho }\;$$

(4) $$N\;=1/(R_{H}e)$$

where RH is the Hall coefficient, and ρ the electrical resistivity.

For the SnO_2_ film cured at 200, 280, and 350 °C, we observe an increase in the carrier concentration from 1.37 ± 0.19 to 4.28 ± 0.54 to 4.47 ± 1.20 × 10^18^ cm^−3^. The Hall mobility also increases from 1.56 ± 0.32 to 1.79 ± 0.67 and to 2.28 ± 0.92 cm^2^/Vs. The mobility increases with the carrier concentration as for oxide semiconductors which has been explained by the percolation conduction of the charge carriers [21]. Also, we note that the values match the trend previously reported for SnO_2_ [19].

**Figure 2:**
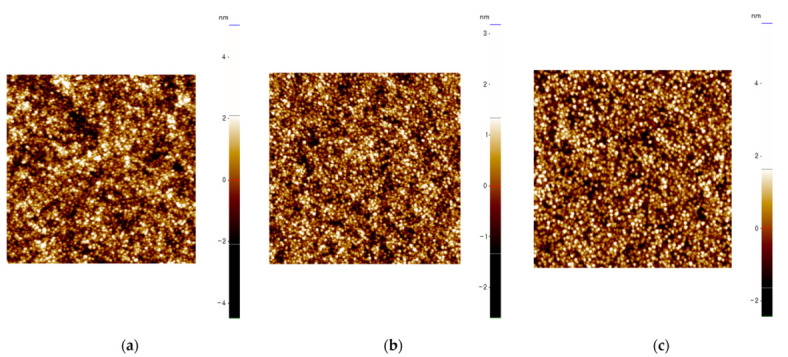
Surface roughness of SnO_2_ as measured by AFM. The thin films were cured at (**a**) 200, (**b**) 280, and (**c**) 350 °C. The color scale is given on the right hand side of each figure.

Figure 2 shows the surface morphologies of SnO_2_ made at the different curing temperatures measured by AFM. The quality of the surface can be assessed by two metrics: the root mean square roughness (R_rms_) and also the peak-to-valley roughness (R_pv_). The respective R_rms_ roughness of SnO_2_ cured at 200 (Figure 2a), 280 (Figure 2b), and 350 °C (Figure 2c) were 1.074, 0.725, and 0.844 nm and their respective R_pv_ values were 9.522, 5.778, and 8.092 nm. Therefore, curing SnO_2_ at 280 °C (Figure 2b) leads to the smoothest surface. We note that we observe the crystallite sizes were in the 10–25 nm range, with the biggest crystallites for the 350 °C cured film. We note that the roughnesses decrease then increase at 280 °C. This was previously reported to be due to the melting of SnCl_2_ at 250 °C [19], which would alter the crystallization of the 280 °C-cured thin films leading to a smoother surface.

### 3.2. Thin Film Transistor and the Origin of Their Hysteresis

**Figure 3:**
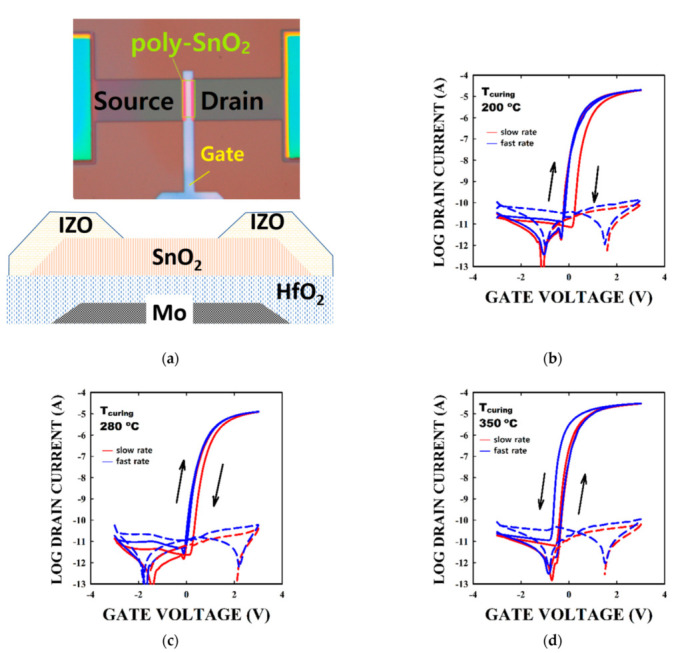
TFT structure and initial IV characteristics. (**a**) the TFT micrograph (**top**) and the TFT structure (**bottom**). The transfer of the poly-SnO_2_ TFTs using a SnO_2_ layer cured at a T_curing_ of (**b**) 200, (**c**) 280, and (**d**) 350 °C, respectively. The solid (dash) line refers to I_DS_ (I_GS_) measured at V_DS_ = 0.1 V. The arrows indicate the direction of the hysteresis.

The typical poly-SnO_2_ TFT structure used in this work is shown at the bottom of Figure 3a. The micrograph of the TFT shown on top of Figure 3a reveals the various elements constituting the TFT. The hysteresis of the TFT transfer curves measured at fast and low rates, when SnO_2_ was cured at 200, 280, and 350 °C are shown in Figure 3b–d, respectively. On average the TFTs made with a SnO_2_ thin film cured at 200, 280, and 350 °C show a linear mobility of 86 ± 12, 90 ± 12, 110 ± 35 cm^2^/Vs; a V_th_ of −0.04 ± 0.05, 0.02 ± 0.12, −0.19 ± 0.14 V; and a subthreshold swing of 103.7 ± 9.9, 112.9 ± 9.4, and 102.7 ± 8.4 mV/dec. We note that the reliability of the extraction of the mobility in particular is highly depending on the size of the TFT [22]. The size chosen in the study should not have a significant impact on the mobility value [15,22]. We gather in Table 1 our present results and various other TFT performances using polycrystalline oxide semiconductors.

We note that even though the hysteresis is an important parameter, the characterization is only seldom reported.

At the T_curing_ of 200 and 280 °C, the hysteresis is clockwise, and the slow sweep rate measurements lead to higher hysteresis than the fast sweep rate measurements. We note that the slow sweep rate hysteresis for the 280 °C-cured-SnO_2_ is smaller than the TFTs with the 200 °C-cured-SnO_2_ layer. Also, the fast sweep measurement rate leads to a ~0.15 V hysteresis. At T_curing_ of 350 °C, an anticlockwise hysteresis is observed, and the fast sweep rate leads to higher hysteresis than the slow sweep rate. The reason why the TFT only shows the anticlockwise hysteresis will be discussed later.

A slow mobility species or a slow phenomenon responsible for the hysteresis for the 200- and 280 °C cured SnO_2_ based TFT could be the reason for the slow sweep measurement rate leading to higher hysteresis than the fast measurement rate [18]. Also, the amount of the species would be smaller in the former than the later. The anticlockwise hysteresis is usually resulting from moving ions in the dielectric, from charge carriers entering the dielectric, or from the polarization of the dielectric. An anticlockwise hysteresis resulting from the polarization of the dielectric would have appeared in all conditions, and cannot therefore explain the behavior of our TFTs. Also, a clockwise hysteresis usually results from charge carriers near/at the channel/dielectric interface. To clarify the underlying phenomenon, we performed PBS and NBS on the TFTs, and measured the hysteresis.

### 3.3. Bias Stress Effect on Poly-SnO_2_ Thin-Film Transistors

**Figure 4:**
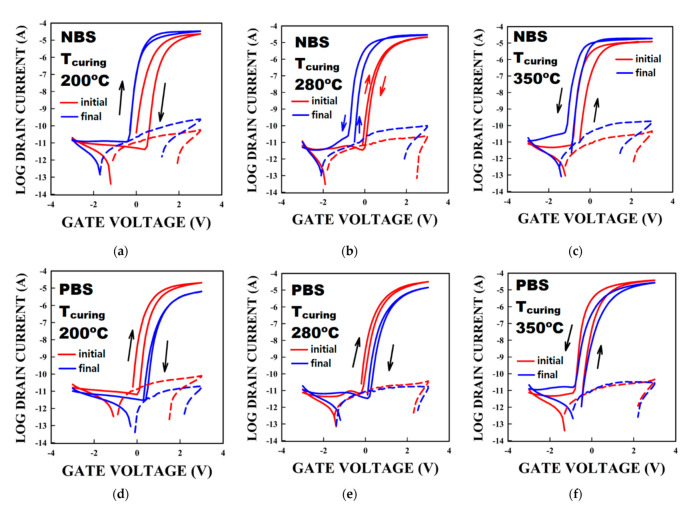
Evolution of the hysteresis curve of SnO_2_ TFT under negative and positive bias stresses for a typical TFT with a SnO_2_ layer made at a T_curing_ of (**a**,**d**) 200, (**b**,**e**) 280, (**c**,**f**) 350 °C, respectively. The solid (dash) line refers to I_DS_ (I_GS_) measured at V_DS_ = 0.1 V. Black arrows indicate the direction of the hysteresis in all graphs except in (**b**) where the red (blue) arrows indicate the direction of the hysteresis at the beginning (the end) of the stress. All stresses were during 3600 s. All hysteresis curves were measured under slow rates.

Figure 4a–f show the variation in the hysteresis under NBS (PBS) for TFT using SnO_2_ having T_curing_ of 200 (Figure 4a,d), 280 (Figure 4b,e), and 350 °C (Figure 4c,f), respectively. As shown in Figure 4a–c, all curves shift negatively under NBS. Interestingly, the direction in the hysteresis changes in the 280 °C-cured SnO_2_ based TFT from clockwise to anticlockwise (Figure 4b). Besides, the hysteresis becomes close to 0 V for the 200 °C-cured SnO_2_ TFT. Not only did we consider the change in the transfer curve (the I_DS_ curve), we also considered the evolution of the gate leakage current (the I_GS_ curve). Under NBS, I_GS_ increases by almost an order of magnitude in all TFTs. The anticlockwise hysteresis appearing under NBS suggests that holes could enter the dielectric during the stress. 

Figure 4d,e show that the 200- and 280 °C-cured SnO_2_ TFT have a positive shift and a decrease in the current under PBS. Also, we observe the decrease of I_GS_ in Figure 4d,e. The decrease in the I_GS_ is almost one order of magnitude for the 200 °C-cured TFT. For the 350 °C-cured TFT, we observe that the TFT current decreases without a significant change in the V_th_. Therefore, considering the various stresses and the various change in V_th_ and the change in I_GS_, we understand that charge carriers are injected from and to the dielectric.

We therefore evaluated the band offsets between SnO_2_ and HfO_2_ for all three different curing temperatures of SnO_2_ [26]. The valence band offset ΔE_v_ is defined as

ΔEv = (E_Hf4f_ − E_VBM_)_HfO2surface_ − (E_Sn3d5/2_ − E_VBM_)_SnO2surface_ − (E_Hf4f_ − E_Sn3d5/2_)_SnO2/HfO2_, (5)

where HfO_2surface_, SnO_2surface_ and SnO_2_/HfO_2_ denote, respectively, the top of the HfO_2_ layer without SnO_2_ on top of it, the top of the SnO_2_ layer, and the HfO_2_/SnO_2_ interface.

**Figure 5:**
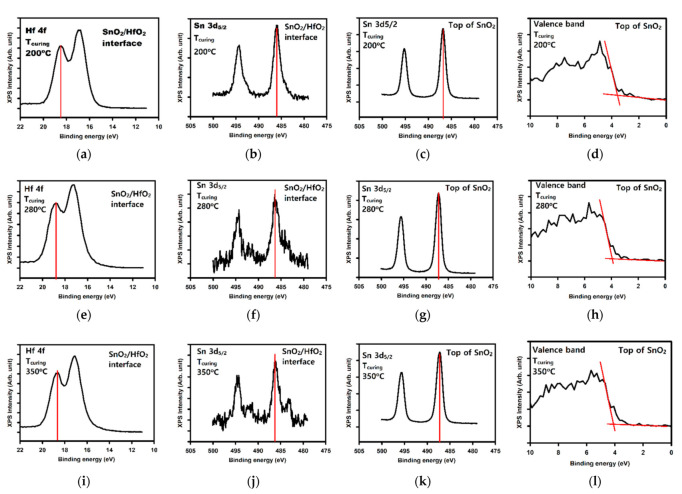
XPS analysis used for the calculation of the band offsets for the various SnO_2_ curing temperatures. (**a**,**e**,**i**) the Hf_4f_ at the interface of SnO_2_/HfO_2_, (**b**,**f**,**j**) the Sn^3^d_5/2_ peak at the SnO_2_/HfO_2_ interface, (**c**,**g**,**k**) the Sn^3^d_5/2_ at the top of the SnO_2_ surface, (**d**,**h**,**l**) the valence band of SnO_2_ at the top of the SnO_2_ surface. The first second, and third line shows the peaks for the SnO_2_ thin film cured at 200, 280, and 350 °C respectively. The peaks and curing conditions are indicated within the figures. The red lines in each graph shows the value of the peak extracted and used for the calculation of ΔE_v_.

Therefore, for each curing temperature we extracted the following peak values: the Hf 4f peak at the SnO_2_/HfO_2_ interface (as shown in Figure 5a,e,i), the Sn 3d_5/2_ peak at the SnO_2_/HfO_2_ interface (as shown in Figure 5b,f,j), the Sn 3d_5/2_ peak on the top of the SnO_2_ layer (Figure 5c,g,k), and the valence band at the top of the SnO_2_ layer (as shown in Figure 5d,h,l). The various extracted values are gathered in Table 2. We note that the values for the position peaks taken for HfO_2_ without SnO_2_ on top are taken from a previous report [16] and we consider the Hf 4f peak position E_Hf4f_ = 18.15 eV, the bandgap of HfO_2_ Eg_HfO2_ = 5.34 eV, and the position of the valence band E_VBM_ = 2.4 eV.

We could evaluate that the valence band offset was 0.17, 0.19 and 0.09 eV for the 200- 280- and 350 °C-cured SnO_2_ layer, respectively. To avoid charge carrier injection, the offset value should be bigger than 1 eV [27]. So, our TFTs having a smaller band offset could have holes injected into the dielectric. 

The small offset can therefore explain the possibility of holes to be trapped into (detrapped from) the dielectric under NBS (PBS). The presence of trapped holes in the dielectric would add up to the electric field attracting more electrons in the channel resulting in an anticlockwise hysteresis. Under PBS, holes can exit the dielectric leading to a decrease in the gate leakage, but also a decrease in the electron current. Thus, trapping of electrons would lead to the observed clockwise hysteresis.

The fact that only the 350 °C-cured SnO_2_ TFT demonstrate the anticlockwise hysteresis could result from the higher density of holes in the SnO_2_ layer compared to the other temperature cured SnO_2_ layer based TFTs. Also, the TFT cured at 350 °C showed an apparent higher mobility. But this value is certainly due to the presence of injected holes in the gate insulator increasing the electron density in the channel during operation and therefore leading to an increased value of the mobility. We note that the 280 °C-cured-SnO_2_ layer showing the smallest R_RMS_ and R_pp_ value leads to the TFT with the smallest clockwise hysteresis. As mentioned before, we previously studied the fabrication of SnO_2_ thin films at various curing and annealing temperatures [19]. We demonstrated that the melting of the precursors at 250 °C had an impact on the various properties of the thin films. The various films had an increase of the R_rms_ roughness and R_PV_ for temperatures higher than the melting temperature. The TFT properties are consistent with the thin film fabrication process and their properties.

**Figure 6:**
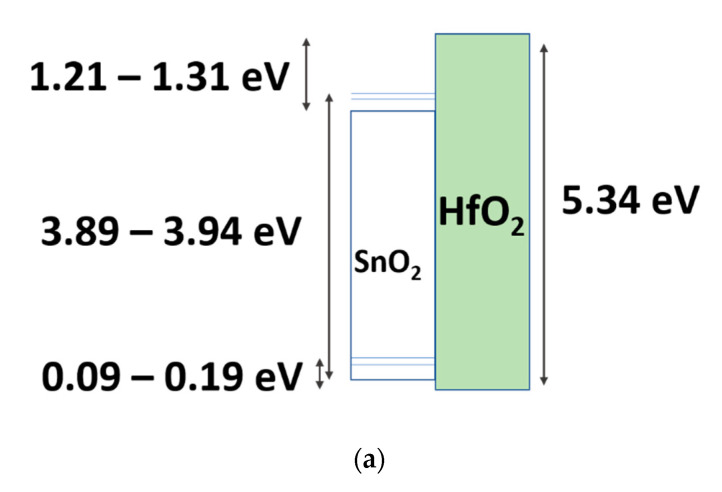

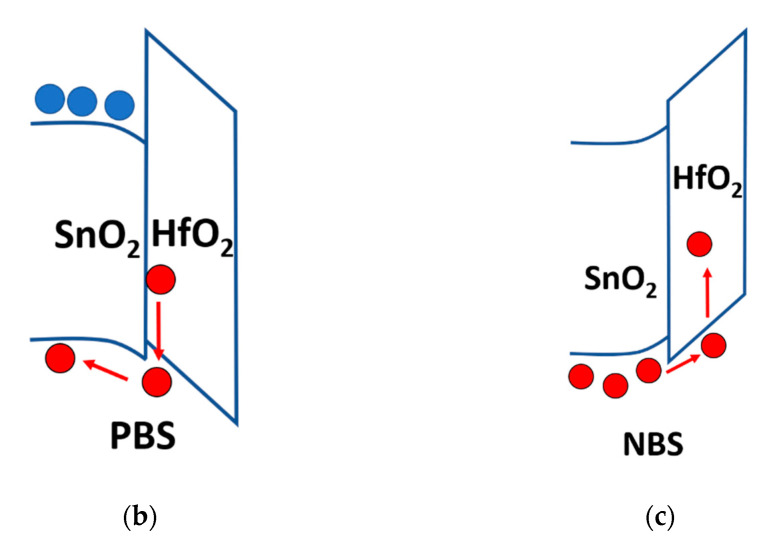
Band offsets of polycrystalline SnO_2_ and HfO_2_. (**a**) Measured band offsets by XPS, (**b**) hole injection into the SnO_2_ layer under PBS, (**c**) hole injection into the HfO_2_ layer under NBS.

Also, we note that holes should be moving slowly in SnO_2_, as they would in IGZO with a mobility of ~0.01 cm^2^/Vs [28]. Under NBS, holes may be injected from the SnO_2_ layer to the dielectric and be trapped, resulting in a negative shift and the anticlockwise hysteresis. This also explains the change of the hysteresis direction for the 280 °C-cured SnO_2_ based TFT shown in Figure 4b. Therefore, we propose that the main phenomenon responsible for the hysteresis in our solution-processed SnO_2_ TFT is the trapping of holes in the dielectric. Detrapping or trapping of holes would therefore monitor the hysteresis. Figure 6 shows the band offsets between HfO_2_ and polycrystalline SnO_2_ Figure 6a summarizes the bandgaps (3.89–3.94 eV for SnO_2_, 5.34 eV for HfO_2_), ΔE_v_ (0.09–0.19 eV), and ΔEc (deduced from the previous values). To find ΔE_v,_ we used the values taken from Figure 5, and gathered in Table 2. Figure 6b,c summarize the proposed mechanism of the hole extraction (injection) during PBS (NBS).

### 3.4. Application to Circuits: Inverters and Ring Oscillators

**Figure 7:**
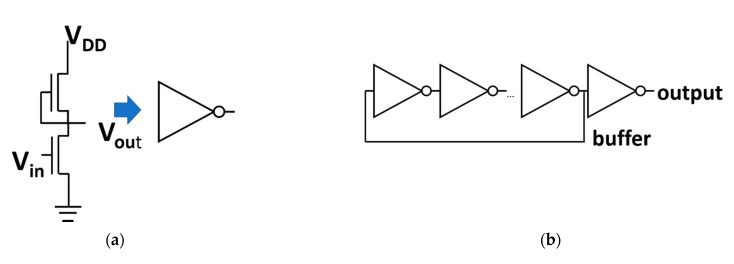

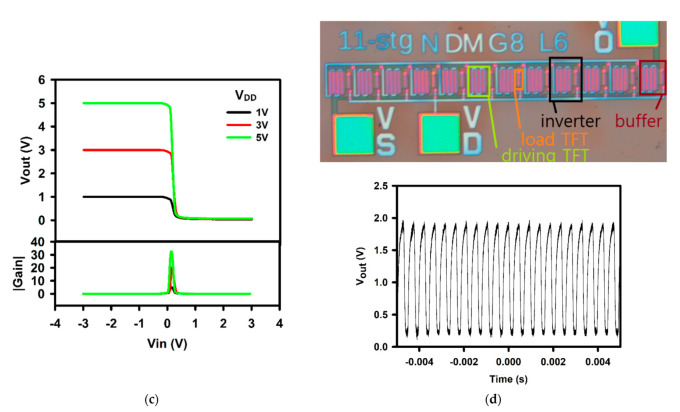
Circuits fabricated with solution processed polycrystalline SnO_2_ TFTs. The schematic of (**a**) an inverter and (**b**) a ring oscillator (R.O.). (**c**) The output curve of an inverter (**top**) and its gain (**bottom**). (**d**) The optical image (**top**) and the output curve of a R.O.

We fabricated both inverters and ring oscillators as circuits to demonstrate the possibility to incorporate SnO_2_ TFT in more advanced circuitry. We chose the devices with a curing step at 280 °C. Indeed, the TFTs showed the clockwise hysteresis. Compared to the 200 °C cured TFTs, the 280 °C-cured TFTs demonstrated smaller hysteresis and higher mobility. We note that the ring oscillators using the TFTs with the anticlockwise hysteresis could not show any oscillation. Figure 7a,b show the respective schematics of an inverter and a ring oscillator (R.O.). The inverter output is shown in Figure 7c. At 5 V the gain is ~30 V/V. The top of Figure 7d shows the optical image of a fabricated ring oscillator, and its various components. In the figure, we indicated the basic inverter structure, but also the buffer. The bottom of Figure 7d shows the output of the R.O. at a V_dd_ of 3 V. The peak-to-peak voltage (V_pp_) is 1.862 V, the frequency is 2.12 kHz. Even though the operating frequency is rather low, which could be due to the low sheet resistance of IZO (~20 ohm/□), and the non-optimized ratio of the TFTs, the present results demonstrate the possibility to further include poly-SnO_2_ TFTs in other more advanced circuitry.

## 4. Conclusions

We successfully fabricated solution processed polycrystalline SnO_2_ TFTs. The SnO_2_ thin films were fabricated at various curing temperature to obtain various carrier concentrations, ranging from ~10^18^ to ~4 × 10^18^ cm^−3^. The TFTs demonstrated a field effect mobility of ~100 cm^2^/Vs. We demonstrated that under stress the hysteresis present in the TFTs was due to the presence of trapped holes in the gate dielectric. We suggest that the trapping occurs due to the small valence band offset between SnO_2_ and HfO_2_. Nonetheless, we demonstrated the possibility of fabricating circuits with SnO_2_ TFTs. The inverters demonstrated a gain of ~30 V/V and the ring oscillators operated at a frequency of 2.12 kHz at a V_DD_ of 3 V. Further optimization of the TFTs by increasing the valence band offset could lead to higher reliability and circuits with higher performances.

## Figures and Tables

**Table 1:** A comparison of various TFT performances using polycrystalline oxide semiconductors and their processing conditions.

Polycrystalline Oxide Semiconductor	Process	Process Temperature (°C)	Gate Insulator	µ (cm^2^/Vs)	Hysteresis (V)	V_th_ (V)	S.S (mV/dec.)	Ref.
CAAC-IGZO	Mist-CVD	450	Al_2_O_3_	90.4	~0	1.5	86	[6]
CAAC-IGZO	Rf-sputtering	300	Al_2_O_3_/HfO_2_/Al_2_O_3_	39.4	N/A	−4.46	380	[7]
In_2_O_3_ *	Spin-coating	250	ZrO_2_	59.8 *	N/A	2.02	180	[23]
In_2_O_3_	Atomic layer deposition	300	Al_2_O_3_	41.8	~0.05	−0.8	100	[24]
ZnO	Spray-coating	350	Al_2_O_3_	39.26	N/A	0.58	167	[8]
ZnO	ALD	350	SiO_2_	43.2	N/A	18.7	N/A	[25]
SnO_2_	Solution process	450	Al_2_O_3_	96.4	~1	1.72	260	[13]
SnO_2_	Physical vapor deposition	400	HfO_2_	147	N/A	0.27	110	[9]
SnO_2_	Spin-coating	350	HfO_2_	90	~0.15	0.02	113	This work

* With Li doping.

**Table 2:** Summary of the peak positions used to extract the valence band offset. All peak positions are in eV.

	Top of SnO_2_ Surface			SnO_2_/HfO_2_ Interface			
SnO_2_ T_curing_ (°C)	E_VBM_	E_Sn3d5/2_	E_Sn3d5/2_-E_VBM_	Sn_3d5/2_	E_Hf4f_	E_hf4f_-E_Sn3d5/2_	ΔE_v_
200	3.6 (d)	486.78 (c)	483.18	486.08 (b)	18.48 (a)	−467.6	+0.17
280	3.94 (h)	487 (g)	483.06	486.32 (f)	18.82 (e)	−467.5	+0.19
350	4.08 (l)	487.31 (k)	483.23	486.25 (j)	18.68 (i)	−467.57	+0.09

The letters in the cells correspond to the peak and peak position shown in the letter-designated-subfigure of Figure 5.

## Data Availability

Data is within the text.
